# Crystal structure of 2-amino-7-hy­droxy-4-(4-hy­droxy­phen­yl)-4*H*-chromene-3-carbo­nitrile

**DOI:** 10.1107/S2056989015012815

**Published:** 2015-07-08

**Authors:** Peter N. Horton, Mehmet Akkurt, Shaaban K. Mohamed, Sabry H. H. Younes, Mustafa R. Albayati

**Affiliations:** a School of Chemistry, University of Southampton, Highfield, Southampton SO17 1BJ, England; b Department of Physics, Faculty of Sciences, Erciyes University, 38039 Kayseri, Turkey; c Chemistry and Environmental Division, Manchester Metropolitan University, Manchester M1 5GD, England; d Chemistry Department, Faculty of Science, Minia University, 61519 El-Minia, Egypt; e Chemistry Department, Faculty of Science, Sohag University, 82524 Sohag, Egypt; f Kirkuk University, College of Science, Department of Chemistry, Kirkuk, Iraq

**Keywords:** crystal structure, chromene, hydrogen bonding,

## Abstract

In the title compound, C_16_H_12_N_2_O_3_, the chromene ring system is nearly planar [maximum deviation from the mean plane = 0.057 (1) Å], and is almost perpendicular to the benzene ring, with a dihedral angle of 85.29 (5)°. In the crystal, mol­ecules are linked by classical N—H⋯O, O—H⋯O and O—H⋯N hydrogen bonds, and weak C—H⋯O hydrogen bonds, forming a three-dimensional supra­molecular network. Furthermore, a weak π–π stacking inter­action is observed; the centroid-to-centroid distance is 3.7260 (7) Å.

## Related literature   

For the synthesis and biological activity of mol­ecules having the 2-amino-7-hy­droxy-4-(4-hy­droxy­phen­yl)-4*H*-chromene unit, see: Mohr *et al.* (1975 ▸); Bianchi & Tava (1987 ▸); Khafagy *et al.* (2002 ▸); Hiramoto *et al.* (1997 ▸); Skommer *et al.* (2006 ▸); Gourdeau *et al.* (2004 ▸); Anderson *et al.* (2005 ▸); Wang *et al.* (2000 ▸).
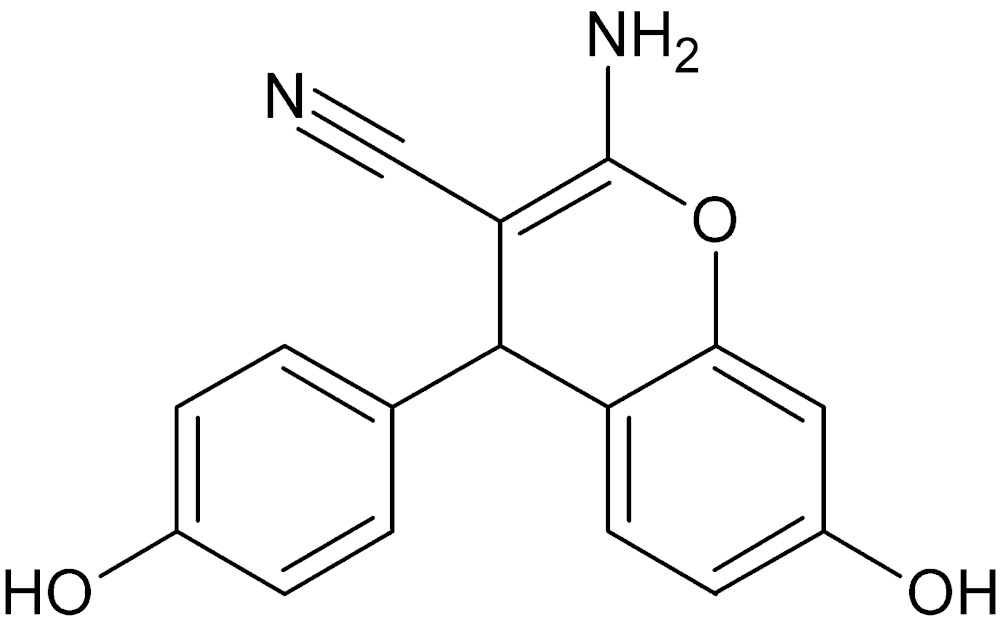


## Experimental   

### Crystal data  

C_16_H_12_N_2_O_3_
*M* *_r_* = 280.28
Monoclinic, 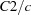

*a* = 18.3084 (13) Å
*b* = 6.0743 (4) Å
*c* = 24.5339 (17) Å
β = 106.471 (2)°
*V* = 2616.5 (3) Å^3^
*Z* = 8
Mo *K*α radiation
μ = 0.10 mm^−1^
*T* = 100 K
0.25 × 0.10 × 0.03 mm

### Data collection  

Rigaku AFC12 (Right) diffractometer
Absorption correction: multi-scan (*CrystalClear-SM*; Rigaku, 2012 ▸) *T*_min_ = 0.830, *T*_max_ = 1.000
13649 measured reflections
2986 independent reflections
2540 reflections with *I* > 2σ(*I*)
*R*_int_ = 0.032

### Refinement  

*R*[*F* ^2^ > 2σ(*F* ^2^)] = 0.034
*wR*(*F* ^2^) = 0.091
*S* = 1.06
2986 reflections
206 parameters
4 restraints
H atoms treated by a mixture of independent and constrained refinement
Δρ_max_ = 0.28 e Å^−3^
Δρ_min_ = −0.20 e Å^−3^

### 

Data collection: *CrystalClear-SM* (Rigaku, 2012 ▸); cell refinement: *CrystalClear-SM*; data reduction: *CrystalClear-SM*; program(s) used to solve structure: *SHELXS97* (Sheldrick, 2008 ▸); program(s) used to refine structure: *SHELXL2014* (Sheldrick, 2015 ▸); molecular graphics: *ORTEP-3 for Windows* (Farrugia, 2012 ▸); software used to prepare material for publication: *WinGX* (Farrugia, 2012 ▸).

## Supplementary Material

Crystal structure: contains datablock(s) global, I. DOI: 10.1107/S2056989015012815/xu5857sup1.cif

Structure factors: contains datablock(s) I. DOI: 10.1107/S2056989015012815/xu5857Isup2.hkl

Supporting information file. DOI: 10.1107/S2056989015012815/xu5857Isup3.cml

**Fig. 1.**
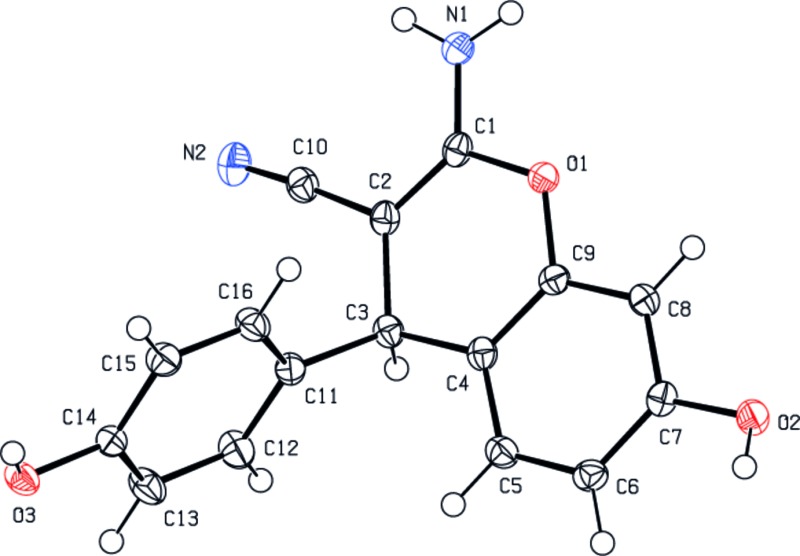
View of the title compound with the atom numbering scheme. Displacement ellipsoids for non-H atoms are drawn at the 50% probability level.

**Fig. 2.**
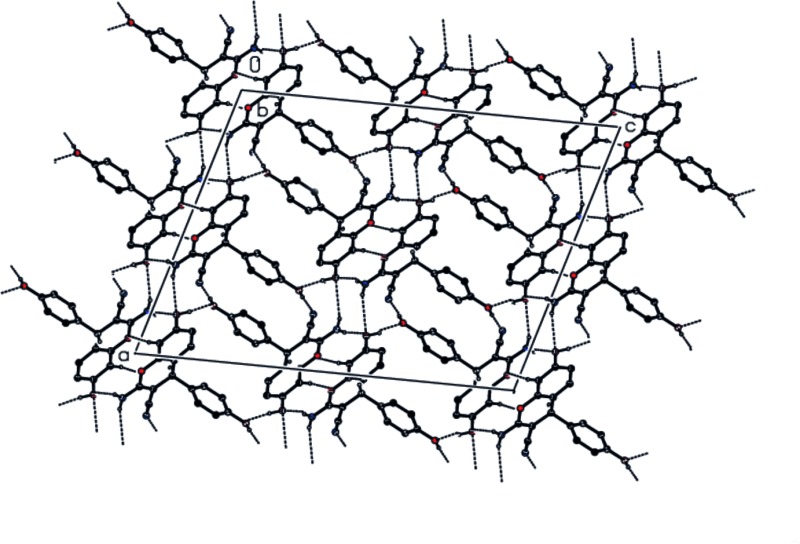
The packing diagram of the title compound viewed down the b axis.

. DOI: 10.1107/S2056989015012815/xu5857fig1.tif
View of the title compound with the atom numbering scheme. Displacement ellipsoids for non-H atoms are drawn at the 50% probability level.

b . DOI: 10.1107/S2056989015012815/xu5857fig2.tif
The packing diagram of the title compound viewed down the *b* axis.

CCDC reference: 1410244

Additional supporting information:  crystallographic information; 3D view; checkCIF report

## Figures and Tables

**Table 1:** Hydrogen-bond geometry (, )

*D*H*A*	*D*H	H*A*	*D* *A*	*D*H*A*
N1H1*N*O2^i^	0.88(1)	2.16(1)	3.0316(14)	169(2)
N1H2*N*O2^ii^	0.90(2)	2.51(2)	3.2052(15)	135(1)
O2H2*O*O3^iii^	0.89(2)	1.81(2)	2.6875(13)	168(2)
O3H3*O*N2^iv^	0.87(1)	1.89(1)	2.7550(14)	174(2)
C8H8O1^i^	0.95	2.47	3.4011(14)	165

Symmetry codes: (i) 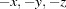; (ii) 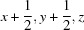; (iii) 
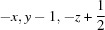
; (iv) 
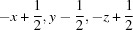
.

## supplementary crystallographic information

### S1. Comment

Heterocycles containing the chromene moiety show interesting biological activities such as antitumor (Mohr *et al.*, 1975), sex pheromone (Bianchi & Tava, 1987), antimicrobial (Khafagy *et al.*, 2002), and mutagenicity (Hiramoto *et al.*, 1997). Interestingly, 2-amino-4*H*-chromene derivatives arises from their potential application in the treatment of human inflammatory TNFa-mediated diseases, such as psoriatic arthritis and rheumatoid and in cancer therapy (Skommer *et al.*, 2006; Gourdeau *et al.*, 2004; Anderson *et al.*, 2005; Wang *et al.*, 2000). Such facts inspired us to synthesize and determine the crystal structure of the title compound in this study.

As shown in Fig. 1, the 4*H*-chromene ring system (O1/C1–C9) of the title compound is almost planar with the puckering parameters of Q(2) = 0.0759 (12) Å and φ(2) = 155.5 (9) °. It makes a dihedral angle of 85.29 (5)° with the C11–C16 phenyl ring.

In the crystal structure, N—H···O, O—H···O, O—H···N and C—H···O hydrogen bonds link the adjacent molecules, forming the three dimensional network (Table 1, Fig. 2). In addition, a weak π-π stacking interaction [*Cg*1···*Cg*1 = 3.7260 (7) Å]; where *Cg*1 is the centroid of the O1/C1–C4/C9 ring of the 4*H*-chromene ring system (O1/C1–C9)] are observed.

### S2. Experimental

A mixture of 1 mmol (180 mg) of 4-hydroxybenzylidene-malononitrile and 1 mmol (110 mg) of resorcinol was refluxed in 10 ml e thanol for 3 h in the presence of few catalytic drops of pipredine. The mixture was cooled at ambient temperature and the resulting solid was filtered off, dried under vacuum and recrytallized from ethanol to furnish white crystals in a good quality suitable for X-ray diffraction. Mp 523 K.

### S3. Refinement

The H atoms of the OH and NH_2_ groups were located in a difference Fourier map and were refined freely [O2—H2O = 0.893 (17) Å, O3—H3O = 0.866 (14) Å, N1—H1N = 0.881 (13) Å and N1—H2N = 0.895 (17) Å]. The H atoms attached to the C atoms were positioned geometrically, with C—H = 0.95 Å and C—H = 1.00 Å for aromatic and methine H, respectively and H atoms were constrained to ride on their parent atoms, with *U*_iso_(H) = 1.2*U*_eq_(C).

### Crystal data

**Table:**

C_16_H_12_N_2_O_3_	*F*(000) = 1168
*M**_r_* = 280.28	*D*_x_ = 1.423 Mg m^−^^3^
Monoclinic, *C*2/*c*	Mo *K*α radiation, λ = 0.71075 Å
Hall symbol: -C 2yc	Cell parameters from 14157 reflections
*a* = 18.3084 (13) Å	θ = 2.3–27.5°
*b* = 6.0743 (4) Å	µ = 0.10 mm^−^^1^
*c* = 24.5339 (17) Å	*T* = 100 K
β = 106.471 (2)°	Blade, brown
*V* = 2616.5 (3) Å^3^	0.25 × 0.10 × 0.03 mm
*Z* = 8	

### Data collection

**Table:**

Rigaku AFC12 (Right) diffractometer	2986 independent reflections
Radiation source: Rotating Anode	2540 reflections with *I* > 2σ(*I*)
Detector resolution: 28.5714 pixels mm^-1^	*R*_int_ = 0.032
profile data from ω–scans	θ_max_ = 27.5°, θ_min_ = 3.3°
Absorption correction: multi-scan (*CrystalClear-SM*; Rigaku, 2012)	*h* = −23→18
*T*_min_ = 0.830, *T*_max_ = 1.000	*k* = −7→7
13649 measured reflections	*l* = −31→31

### Refinement

**Table:**

Refinement on *F*^2^	4 restraints
Least-squares matrix: full	Hydrogen site location: difference Fourier map
*R*[*F*^2^ > 2σ(*F*^2^)] = 0.034	H atoms treated by a mixture of independent and constrained refinement
*wR*(*F*^2^) = 0.091	*w* = 1/[σ^2^(*F*_o_^2^) + (0.0397*P*)^2^ + 1.7337*P*] where *P* = (*F*_o_^2^ + 2*F*_c_^2^)/3
*S* = 1.06	(Δ/σ)_max_ < 0.001
2986 reflections	Δρ_max_ = 0.28 e Å^−^^3^
206 parameters	Δρ_min_ = −0.20 e Å^−^^3^

### Special details

**Table:**

Geometry. Bond distances, angles *etc*. have been calculated using the rounded fractional coordinates. All su's are estimated from the variances of the (full) variance-covariance matrix. The cell e.s.d.'s are taken into account in the estimation of distances, angles and torsion angles
Refinement. Refinement on *F*^2^ for ALL reflections except those flagged by the user for potential systematic errors. Weighted *R*-factors *wR* and all goodnesses of fit *S* are based on *F*^2^, conventional *R*-factors *R* are based on *F*, with *F* set to zero for negative *F*^2^. The observed criterion of *F*^2^ > σ(*F*^2^) is used only for calculating -*R*-factor-obs *etc*. and is not relevant to the choice of reflections for refinement. *R*-factors based on *F*^2^ are statistically about twice as large as those based on *F*, and *R*-factors based on ALL data will be even larger.

### Fractional atomic coordinates and isotropic or equivalent isotropic displacement parameters (Å^2^)

**Table:**

	*x*	*y*	*z*	*U*_iso_*/*U*_eq_	
O1	0.05989 (4)	0.30160 (13)	0.03455 (3)	0.0187 (2)	
O2	−0.15605 (5)	−0.03145 (14)	0.06613 (4)	0.0226 (3)	
O3	0.20866 (5)	0.79736 (15)	0.35127 (3)	0.0213 (3)	
N1	0.16338 (6)	0.41872 (18)	0.01319 (4)	0.0207 (3)	
N2	0.21498 (6)	0.93998 (18)	0.09021 (5)	0.0268 (3)	
C1	0.11540 (6)	0.45728 (19)	0.04464 (5)	0.0173 (3)	
C2	0.11794 (6)	0.62821 (19)	0.08107 (5)	0.0178 (3)	
C3	0.06229 (6)	0.66058 (19)	0.11588 (5)	0.0174 (3)	
C4	0.00672 (6)	0.46994 (19)	0.10543 (5)	0.0170 (3)	
C5	−0.04921 (6)	0.4552 (2)	0.13396 (5)	0.0192 (3)	
C6	−0.10354 (6)	0.2906 (2)	0.12215 (5)	0.0196 (3)	
C7	−0.10248 (6)	0.13336 (19)	0.08098 (5)	0.0180 (3)	
C8	−0.04727 (6)	0.14161 (19)	0.05235 (5)	0.0172 (3)	
C9	0.00636 (6)	0.30919 (19)	0.06543 (5)	0.0164 (3)	
C10	0.17293 (7)	0.7951 (2)	0.08532 (5)	0.0200 (3)	
C11	0.10251 (6)	0.69419 (19)	0.17886 (5)	0.0178 (3)	
C12	0.08736 (7)	0.8801 (2)	0.20677 (5)	0.0235 (3)	
C13	0.12262 (7)	0.9120 (2)	0.26446 (5)	0.0250 (3)	
C14	0.17396 (6)	0.7573 (2)	0.29446 (5)	0.0182 (3)	
C15	0.19056 (7)	0.5711 (2)	0.26720 (5)	0.0206 (3)	
C16	0.15444 (7)	0.5408 (2)	0.20975 (5)	0.0210 (3)	
H1N	0.1546 (9)	0.304 (2)	−0.0097 (6)	0.034 (4)*	
H2N	0.2042 (8)	0.505 (3)	0.0173 (7)	0.034 (4)*	
H2O	−0.1713 (10)	−0.071 (3)	0.0962 (7)	0.049 (5)*	
H3	0.03240	0.79710	0.10170	0.0210*	
H3O	0.2327 (9)	0.681 (2)	0.3673 (7)	0.040 (5)*	
H5	−0.04990	0.56130	0.16230	0.0230*	
H6	−0.14130	0.28470	0.14190	0.0240*	
H8	−0.04620	0.03470	0.02430	0.0210*	
H12	0.05240	0.98710	0.18620	0.0280*	
H13	0.11150	1.03930	0.28310	0.0300*	
H15	0.22620	0.46570	0.28770	0.0250*	
H16	0.16540	0.41290	0.19120	0.0250*	

### Atomic displacement parameters (Å^2^)

**Table:**

	*U*^11^	*U*^22^	*U*^33^	*U*^12^	*U*^13^	*U*^23^
O1	0.0184 (4)	0.0189 (4)	0.0203 (4)	−0.0043 (3)	0.0080 (3)	−0.0035 (3)
O2	0.0241 (4)	0.0258 (5)	0.0199 (4)	−0.0092 (3)	0.0096 (3)	−0.0028 (3)
O3	0.0241 (4)	0.0233 (5)	0.0150 (4)	0.0059 (3)	0.0030 (3)	−0.0014 (3)
N1	0.0188 (5)	0.0219 (5)	0.0228 (5)	−0.0046 (4)	0.0081 (4)	−0.0032 (4)
N2	0.0253 (5)	0.0250 (6)	0.0279 (6)	−0.0066 (4)	0.0039 (4)	−0.0015 (4)
C1	0.0157 (5)	0.0183 (6)	0.0164 (5)	−0.0015 (4)	0.0019 (4)	0.0028 (4)
C2	0.0177 (5)	0.0186 (6)	0.0157 (5)	−0.0021 (4)	0.0026 (4)	0.0009 (4)
C3	0.0188 (5)	0.0162 (5)	0.0161 (5)	0.0007 (4)	0.0031 (4)	0.0001 (4)
C4	0.0168 (5)	0.0172 (6)	0.0153 (5)	0.0011 (4)	0.0016 (4)	0.0015 (4)
C5	0.0206 (5)	0.0203 (6)	0.0164 (5)	0.0021 (4)	0.0047 (4)	−0.0009 (4)
C6	0.0186 (5)	0.0234 (6)	0.0176 (5)	0.0013 (4)	0.0064 (4)	0.0021 (4)
C7	0.0174 (5)	0.0182 (6)	0.0173 (5)	−0.0020 (4)	0.0032 (4)	0.0024 (4)
C8	0.0192 (5)	0.0168 (5)	0.0151 (5)	0.0001 (4)	0.0040 (4)	−0.0002 (4)
C9	0.0153 (5)	0.0188 (6)	0.0149 (5)	0.0010 (4)	0.0040 (4)	0.0021 (4)
C10	0.0208 (6)	0.0212 (6)	0.0165 (5)	0.0004 (5)	0.0030 (4)	0.0000 (4)
C11	0.0176 (5)	0.0187 (6)	0.0166 (5)	−0.0017 (4)	0.0042 (4)	−0.0009 (4)
C12	0.0258 (6)	0.0205 (6)	0.0213 (6)	0.0055 (5)	0.0022 (5)	−0.0009 (5)
C13	0.0299 (6)	0.0215 (6)	0.0217 (6)	0.0065 (5)	0.0044 (5)	−0.0048 (5)
C14	0.0177 (5)	0.0213 (6)	0.0158 (5)	−0.0009 (4)	0.0052 (4)	−0.0013 (4)
C15	0.0203 (5)	0.0209 (6)	0.0194 (6)	0.0049 (4)	0.0039 (4)	0.0012 (5)
C16	0.0235 (6)	0.0191 (6)	0.0198 (6)	0.0034 (4)	0.0053 (5)	−0.0028 (4)

### Geometric parameters (Å, º)

**Table:**

O1—C1	1.3586 (14)	C6—C7	1.3943 (17)
O1—C9	1.3992 (14)	C7—C8	1.3857 (16)
O2—C7	1.3763 (15)	C8—C9	1.3873 (16)
O3—C14	1.3796 (14)	C11—C12	1.3888 (17)
N1—C1	1.3440 (16)	C11—C16	1.3921 (17)
N2—C10	1.1529 (17)	C12—C13	1.3926 (17)
O2—H2O	0.893 (17)	C13—C14	1.3842 (17)
O3—H3O	0.866 (14)	C14—C15	1.3907 (17)
N1—H1N	0.881 (13)	C15—C16	1.3890 (17)
C1—C2	1.3622 (16)	C3—H3	1.0000
N1—H2N	0.895 (17)	C5—H5	0.9500
C2—C3	1.5164 (16)	C6—H6	0.9500
C2—C10	1.4116 (17)	C8—H8	0.9500
C3—C11	1.5252 (17)	C12—H12	0.9500
C3—C4	1.5146 (16)	C13—H13	0.9500
C4—C9	1.3831 (16)	C15—H15	0.9500
C4—C5	1.3975 (16)	C16—H16	0.9500
C5—C6	1.3819 (17)		
			
C1—O1—C9	118.75 (9)	C3—C11—C16	121.40 (10)
C7—O2—H2O	110.1 (12)	C12—C11—C16	118.48 (11)
C14—O3—H3O	110.1 (10)	C3—C11—C12	120.12 (10)
O1—C1—N1	110.55 (10)	C11—C12—C13	120.96 (11)
C1—N1—H1N	118.2 (11)	C12—C13—C14	119.68 (11)
C1—N1—H2N	119.6 (11)	O3—C14—C13	117.79 (11)
H1N—N1—H2N	122.1 (15)	C13—C14—C15	120.29 (11)
O1—C1—C2	122.33 (10)	O3—C14—C15	121.90 (11)
N1—C1—C2	127.11 (11)	C14—C15—C16	119.32 (11)
C3—C2—C10	116.38 (10)	C11—C16—C15	121.26 (11)
C1—C2—C3	124.40 (10)	C2—C3—H3	107.00
C1—C2—C10	119.13 (11)	C4—C3—H3	107.00
C4—C3—C11	112.46 (10)	C11—C3—H3	108.00
C2—C3—C11	112.30 (10)	C4—C5—H5	119.00
C2—C3—C4	109.38 (9)	C6—C5—H5	119.00
C3—C4—C5	121.11 (10)	C5—C6—H6	120.00
C5—C4—C9	116.96 (11)	C7—C6—H6	120.00
C3—C4—C9	121.85 (10)	C7—C8—H8	121.00
C4—C5—C6	121.83 (11)	C9—C8—H8	121.00
C5—C6—C7	119.37 (11)	C11—C12—H12	120.00
O2—C7—C8	117.36 (10)	C13—C12—H12	120.00
C6—C7—C8	120.29 (11)	C12—C13—H13	120.00
O2—C7—C6	122.33 (10)	C14—C13—H13	120.00
C7—C8—C9	118.65 (11)	C14—C15—H15	120.00
O1—C9—C4	122.98 (10)	C16—C15—H15	120.00
O1—C9—C8	114.15 (10)	C11—C16—H16	119.00
C4—C9—C8	122.88 (11)	C15—C16—H16	119.00
N2—C10—C2	175.64 (14)		
			
C9—O1—C1—N1	177.24 (9)	C5—C4—C9—O1	−178.77 (10)
C9—O1—C1—C2	−4.25 (16)	C5—C4—C9—C8	1.38 (18)
C1—O1—C9—C8	−179.32 (10)	C3—C4—C9—C8	−175.44 (11)
C1—O1—C9—C4	0.81 (16)	C3—C4—C5—C6	175.56 (11)
N1—C1—C2—C3	−179.28 (11)	C4—C5—C6—C7	0.50 (18)
N1—C1—C2—C10	4.29 (19)	C5—C6—C7—C8	0.26 (18)
O1—C1—C2—C10	−173.96 (10)	C5—C6—C7—O2	−178.29 (11)
O1—C1—C2—C3	2.47 (18)	O2—C7—C8—C9	178.43 (10)
C10—C2—C3—C4	178.86 (10)	C6—C7—C8—C9	−0.19 (18)
C1—C2—C3—C4	2.34 (16)	C7—C8—C9—C4	−0.67 (18)
C1—C2—C3—C11	127.93 (12)	C7—C8—C9—O1	179.46 (10)
C10—C2—C3—C11	−55.55 (14)	C3—C11—C12—C13	178.99 (11)
C2—C3—C4—C9	−5.55 (15)	C16—C11—C12—C13	−0.61 (19)
C11—C3—C4—C5	52.26 (15)	C3—C11—C16—C15	−179.53 (11)
C2—C3—C11—C12	124.45 (12)	C12—C11—C16—C15	0.07 (19)
C2—C3—C11—C16	−55.96 (15)	C11—C12—C13—C14	0.48 (19)
C11—C3—C4—C9	−131.05 (12)	C12—C13—C14—O3	178.81 (11)
C2—C3—C4—C5	177.75 (11)	C12—C13—C14—C15	0.21 (19)
C4—C3—C11—C16	67.94 (14)	O3—C14—C15—C16	−179.28 (11)
C4—C3—C11—C12	−111.66 (12)	C13—C14—C15—C16	−0.74 (19)
C9—C4—C5—C6	−1.29 (17)	C14—C15—C16—C11	0.60 (19)
C3—C4—C9—O1	4.41 (18)		

### Hydrogen-bond geometry (Å, º)

**Table:**

*D*—H···*A*	*D*—H	H···*A*	*D*···*A*	*D*—H···*A*
N1—H1*N*···O2^i^	0.88 (1)	2.16 (1)	3.0316 (14)	169 (2)
N1—H2*N*···O2^ii^	0.90 (2)	2.51 (2)	3.2052 (15)	135 (1)
O2—H2*O*···O3^iii^	0.89 (2)	1.81 (2)	2.6875 (13)	168 (2)
O3—H3*O*···N2^iv^	0.87 (1)	1.89 (1)	2.7550 (14)	174 (2)
C8—H8···O1^i^	0.95	2.47	3.4011 (14)	165

Symmetry codes: (i) −*x*, −*y*, −*z*; (ii) *x*+1/2, *y*+1/2, *z*; (iii) −*x*, *y*−1, −*z*+1/2; (iv) −*x*+1/2, *y*−1/2, −*z*+1/2.
