# Supplementary figures and images for: Crystal structure of 2-amino-7-hy­droxy-4-(4-hy­droxy­phen­yl)-4H-chromene-3-carbo­nitrile
Source: Acta Crystallogr E Crystallogr Commun. 2015 Jul 8;71(Pt 8):o546–7. doi: 10.1107/S2056989015012815 (PMC4571389; doi:10.1107/S2056989015012815)

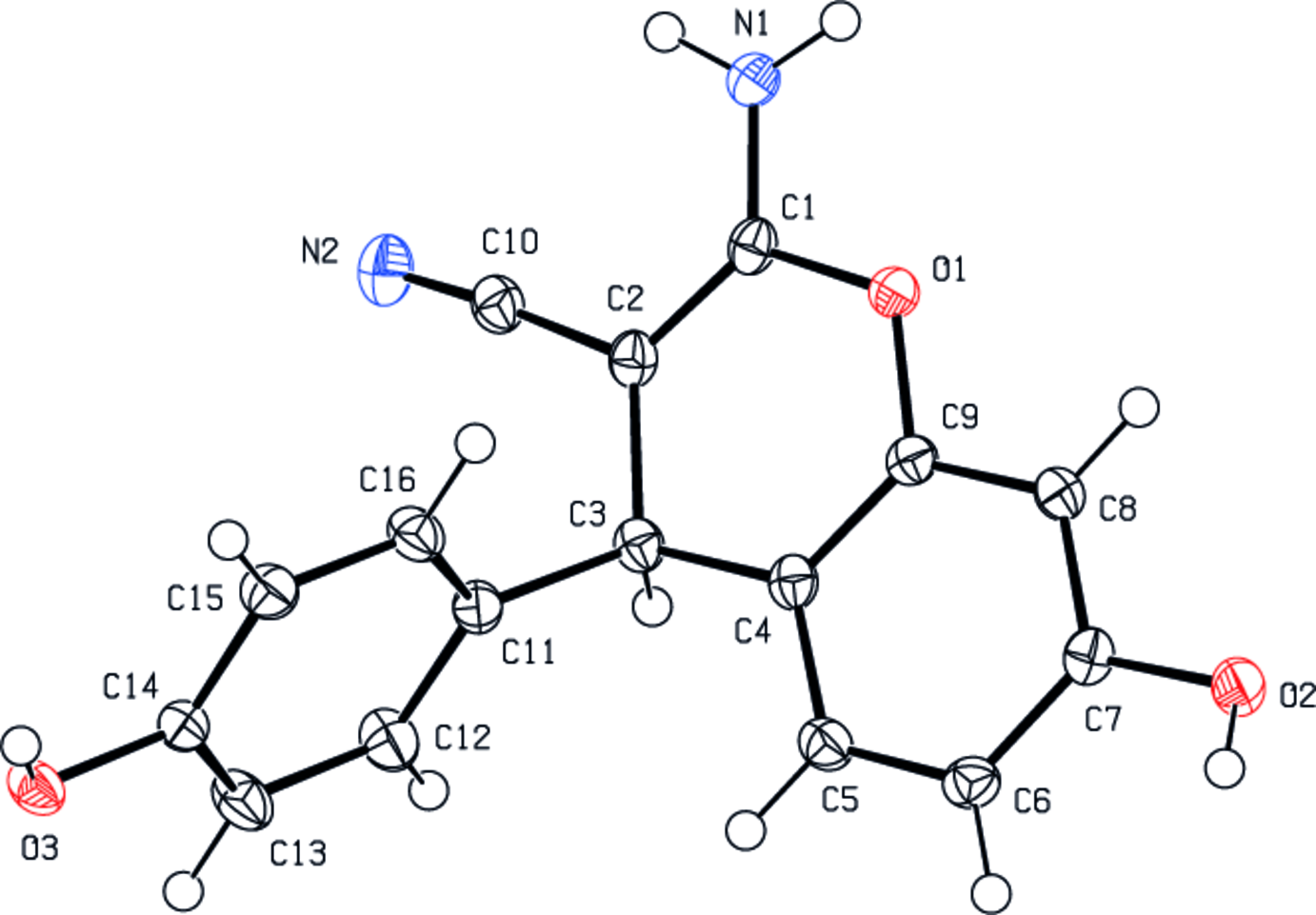

Supplement: Supplementary file 4 [file e-71-0o546-fig1.tif]

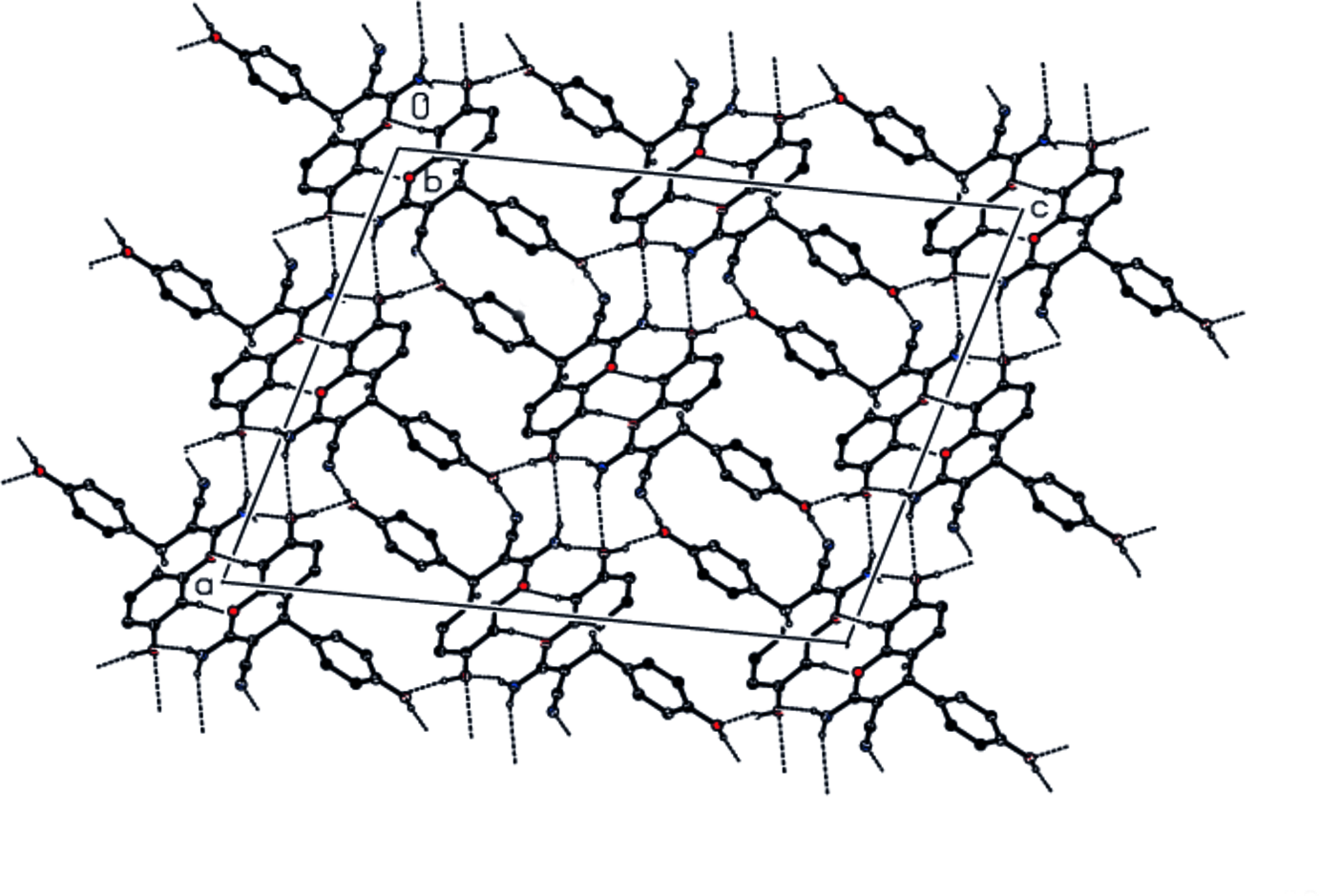

Supplement: Supplementary file 5 [file e-71-0o546-fig2.tif]
